# Association between the incident hypertension duration and cognitive performance in older adults: data from the NHANES 2011–2014

**DOI:** 10.1007/s40520-024-02836-1

**Published:** 2024-08-30

**Authors:** Chunlei Liu, Qi Li, Zhuqing Li, Li Wang, Che Wang, Xiaoyu Du, Wenjuan Song, Xiaotong Sun, Chengzhi Lu

**Affiliations:** 1 School of Medicine, Nankai University, Tianjin, 300071 China; 2 Department of Cardiology, Tianjin First Center Hospital, Tianjin, 300192 China; 3 Department of Cardiology, The First Central Clinical School, Tianjin Medical University, Tianjin, 300192 China

**Keywords:** Hypertension cognition performance vascular cognitive impairment NHANES

## Abstract

**Background:**

Established evidences have demonstrated that hypertension was associated with the cognitive impairment. But the associations between the duration of hypertension exposure and cognitive performance are still inconclusive.

**Objectives:**

The objective of this study was to assess the association between the duration of hypertension diagnosis and cognitive performance in older adults by the National Health and Nutrition Examination Survey (2011–2014).

**Methods:**

To evaluate the relationship between the hypertension duration and cognitive performance, we conducted the logistic regression analysis. Furthermore, we also performed the Restricted cubic spline (RCS) analysis to assess the nonlinear relationship between the duration of exposure to hypertension and cognitive performance.

**Results:**

Initially, total 19,931 participants were included in this study, and 2928 individuals were enrolled. With the increase of hypertension duration, more risk of cognitive impairment was observed in the Digit Symbol Substitution test (DSST) (OR = 1.012, 1.006–1.019), and a similar trend was observed in Animal Fluency test (AFT) (OR = 1.009,1.003–1.016). The RCS results showed that the hypertension duration pattern was linear associated with the risk of cognitive impairment in DDST (P for non–linearity = 0.758). Meanwhile, subgroups analysis of midlife hypertension, we revealed that linear association with the risk of cognitive impairment in DSST (P for non–linearity = 0.391) and CERAD (P for non–linearity = 0.849) among hypertension diagnose < 55 years populations.

**Conclusion:**

Collectively, our finding indicates that longer duration of exposure to hypertension worsens the cognition performance, especially for middle-aged hypertension.

## Introduction

Hypertension is a common cardiovascular disorder with consistently high arterial blood pressure [1, 2]. It is a predominant cause of many cardiovascular diseases, resulting of the heart diseases, strokes, and renal failure, etc. [3, 4]. Hypertension can exert harmful effects through many mechanisms, including enhanced cardiac workload, vascular integrity compromise, cerebral vasculature impairment and other diverse complications [5, 6]. Therefore, it emphasizes in taking the early detection and proactive measures to prevent the damage of hypertension [7]. 

Cognitive function usually refers to how an individual processes external and internal information, and how they utilize this information for thinking and decision-making [8–10]. It covers psychological activities such as perception, attention, memory, thinking, and language, representing a series of complex mental processes [11, 12]. Cognitive dysfunction can result from a variety of factors, including neurodegenerative diseases, mental health issues and vascular cognitive impairment, etc. [13, 14]. The common symptoms of cognitive dysfunction include memory loss, lack of concentration, language barriers and decreased spatial orientation abilities, etc. Such manifestations can significantly affect daily life quality and work proficiency.

Previous studies have revealed the association of hypertension and cognitive function [15, 16]. These evidences are largely derived from epidemiological community studies that identify hypertension as a risk factor for a variety of adverse outcomes, including cognitive decline, mild cognitive impairment (MCI), and dementia [17]. Another prospective longitudinal cohort study also has explored the relationship between high blood pressure (BP) levels and cognitive domains [18]. It suggested that high systolic BP values were significant for faster decline on the Clinical Dementia Rating Sum of Boxes score, which indicated that hypertension aggravated the cognitive declines. Furthermore, an investigation from the Framingham Heart Study Offspring cohort has demonstrated a significant association between the duration of hypertension exposure and the burden of cerebral small vessel disease (CSVD) [19]. Additionally, the duration of hypertension contributed to the adverse results of cognition performance have been explored by some cohort studies [13, 20]. However, one previous study suggested that cognitive decline was irrespective of hypertension duration exposure [21]. Therefore, these results are inconsistent among studies that investigated the effect of hypertension duration on cognitive performance.

Therefore, the study aimed to reconfirmed whether duration of exposure to hypertension has association with cognition decline by a cross-section study from the National Health and Nutrition Examination Survey (NHANES). And we hypothesize that longer duration of exposure to hypertension suggests a greater decline in cognitive.

## Methods

### Study aim and design

The NHANES by the Centers for Disease Control and Prevention (CDC) in partnership with the National Center for Health Statistics (NCHS), is designed to assess the health and nutritional status of the populations across the United States. It includes detailed inquiries into demographic and socioeconomic backgrounds, dietary habits, and health-related issues, as well as laboratory tests. This study, which focuses on individuals using data from the 2011–2012 and 2013–2014 cycles, adheres to strict ethical guidelines and requires informed consent from all participants, having received approval from the NCHS Research Ethics Review Board.

### Study population

**Fig. 1:**
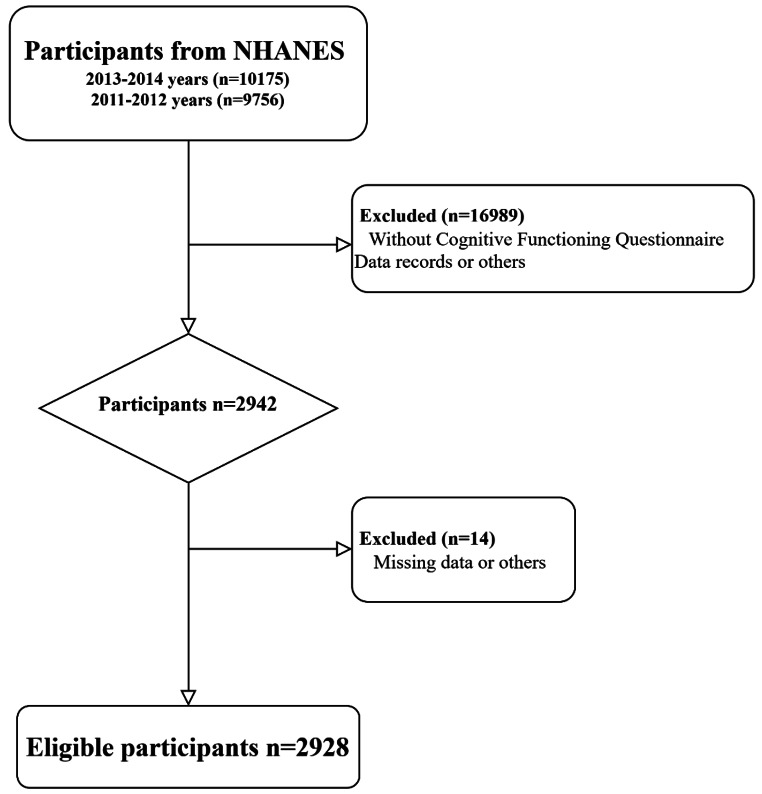
Flowchart of inclusion and exclusion assessment for the eligible participants in the study

A total of 19,931 participants were assessed across the two NHANES cycles. Of these, 2942 underwent cognitive assessment. And after excluding those with missing BP questionnaire data records or others, 2,928 populations were included in the analysis (Fig. 1). The recruitment process was detailed in Fig. 1, and the study protocol was approved by the NCHS Research Ethics Review Board, with all participants providing written informed consent.

### Definition of hypertension

Participants were classified as having hypertension based on the following criteria: a mean systolic blood pressure ≥ 140 mmHg and/or a mean diastolic blood pressure ≥ 90 mmHg, or a self-reported history of taking prescribed antihypertensive medication, or having been told had hypertension by a healthcare professional. The NHANES protocol entails a series of three consecutive blood pressure measurements, with the option for an additional reading to ensure accuracy in diagnosing hypertension.

### The duration of hypertension diagnosis

In the study, the duration of hypertension exposure was assessed as the time interval between the age in years told had hypertension and age at screening in this survey.

### Cognitive assessment

Cognitive function among participants was evaluated through the tests that included the Consortium to Establish a Registry for Alzheimer’s Disease (CERAD) word learning and recall modules, the Animal Fluency test (AFT), and the Digit Symbol Substitution Test (DSST) as part of NHANES. The CERAD test measures the ability to learn and recall new verbal information immediately, the AFT to measure categorical verbal fluency, and the DSST for processing speed and attention. Although there are no established cutoffs for the DSST, CERAD, and AFT to indicate cognitive impairment, this study adopted the 25th percentile of the scores as a benchmark for identifying cognitive dysfunction. In all tests, superior cognitive function represented higher scores.

### Covariates

Additionally, in our analysis, we also adopted different covariates, including demographic data, examination data, laboratory data, and various questionnaire data. The variables comprised of age, gender (male and female), the use of antihypertensive medications, systolic BP, diastolic BP, body mass index (BMI), smoking, alcohol use, diabetes, and lipid profiles, etc.

### Statistical analysis

Statistical analyses in this study were conducted using R version 4.1.3 and SPSS (Statistical Package for the Social Sciences) 24.0 version. Continuous variables were presented as the mean ± standard error (SE), while categorical variables were expressed as frequencies and percentages. For continuous variables, the Student’s t-test was utilized, whereas the chi-square test was employed for categorical variables. Logistic regression models were constructed to explore the relationship between the hypertensive duration and cognitive function. Model 1 was a logistic regression, Model 2 adjusted for diabetes status, Model 3 adjusted for BMI, and Model 4 adjusted for gender, BMI, lipid profiles, smoking, alcohol use, diabetes, and antihypertensive drug use. Additionally, a restricted cubic spline (RCS) was performed to assess the nonlinear relationship between hypertension duration and cognitive performance as well as subgroups analysis. All statistical analyses were appropriately weighted according to the requirements set by the National Center for Health Statistics (NCHS).

## Results

### Characteristics of participants

In this study, based on the NHANES data, total 19,931 participants were included. The characteristics of subjects, categorized by hypertension status, were summarized in Table 1. Of the 2928 enrolled participants, with a mean age of 69.482 years, totally 62.466% individuals had hypertension. Besides, BP examination data revealed that those with hypertension predominantly exhibited elevated systolic BP (*p* = 0.000). It also indicated that hypertensive group tended to be older (*p* = 0.000). And individuals with hypertension were more likely to have diabetic disease, higher BMI, and consume of alcohol and cigarettes (*p* = 0.000). Notably, hypertensive participants inclined to have poor cognitive performance with lower CERAD test, Animal Fluency test and DSST scores (*p*<0.050).

**Table 1:** Baseline characteristics of the study participants

	Overall (no. = 2928)	Hypertension (no. = 1829)	Nonhypertension (no. = 1099)	*p*
Age(years)	69.482(0.125)	70.021(0.160)	68.582(0.199)	0.000
Gender (%)				0.000
Male	1425(48.668)	842(46.036)	583(53.048)	
Female	1503(51.332)	987(53.963)	516(46.952)	
Taking prescription for hypertension (%)				0.000
Yes	1742(59.494)	1742(95.243)		
No	84(2.869)	84(4.593)		
Others	1102(37.636)			
Blood pressure (BP, mmHg)				
Systolic BP (1st)	133.052(19.738)	136.601(0.506)	129.296(0.535)	0.000
Diastolic BP (1st)	68.728(14.028)	68.344(0.364)	69.356(0.393)	0.070
Systolic BP (2nd)	133.05(19.738)	135.732(0.494)	128.614(0.535)	0.000
Diastolic BP (2nd)	67.532(15.056)	67.284(0.367)	67.943(0.430)	0.260
Systolic BP (3th)	132.076(19.600)	134.657(0.490)	127.805(0.535)	0.000
Diastolic BP (3th)	66.964(15.923)	66.673(0.399)	67.443(0.451)	0.216
Doctor told you have diabetes (%)				0.000
Yes	686(23.429)	518(28.321)	168(15.287)	
No	2108(71.995)	1211(66.211)	897(81.620)	
Borderline	132(4.058)	99(5.413)	33(3.003)	
Others	2(0.068)			
Body Mass Index (kg/m^2^)	29.060(6.361)	30.013(0.155)	27.485(0.172)	0.000
HDL-Cholesterol (mmol/L)	1.408(0.424)	1.379(0.009)	1.454(0.013)	0.000
Triglyceride (mmol/L)	1.382(0.806)	1.456(0.028)	1.258(0.0312)	0.000
LDL-cholesterol (mmol/L)	2.855(0.937)	2.761(0.0319)	3.011(0.040)	0.000
Total Cholesterol (mmol/L)	4.950(1.119)	4.842(0.027)	5.128(0.032)	0.000
At least 12 alcohol drinks/lifetime (%)				0.789
Yes	460(15.710)	301(16.457)	159(14.468)	
No	452(15.437)	295(16.129)	157(14.286)	
Others	2016(68.852)			
Do you now smoke cigarettes (%)				0.115
Every day	311(10.626)	179(9.787)	132(12.011)	
Some days	61(2.083)	38(2.078)	23(2.093)	
Not at all	1114(38.046)	713(38.983)	401(36.488)	
Others	1442(49.249)			
**CERAD**				
Score Trial 1 Recall	4.695(0.031)	4.646(0.041)	4.772(0.050)	0.051
Score Trial 2 Recall	6.696(0.034)	6.637(0.042)	6.798(0.056)	0.021
Score Trial 3 Recall	7.528(0.335)	7.449(0.043)	7.660(0.053)	0.002
Score Delayed Recall	5.917(0.043)	5.826(0.054)	6.085(0.070)	0.003
**Animal Fluency: Score Total**	16.564(5.474)	16.054(0.124)	17.427(0.170)	0.000
**Digit Symbol Coding: Score**	45.659(17.313)	44.049(0.399)	48.328(0.525)	0.000

Abbreviations: CRDAD Consortium to Establish a Registry for Alzheimer’s Disease; HDL: High Density Lipoprotein; LDL: Low Density Lipoprotein

Continuous variable-values shown are mean (standard error)

### Association between the duration of exposure to hypertension with cognition function

**Fig. 2:**
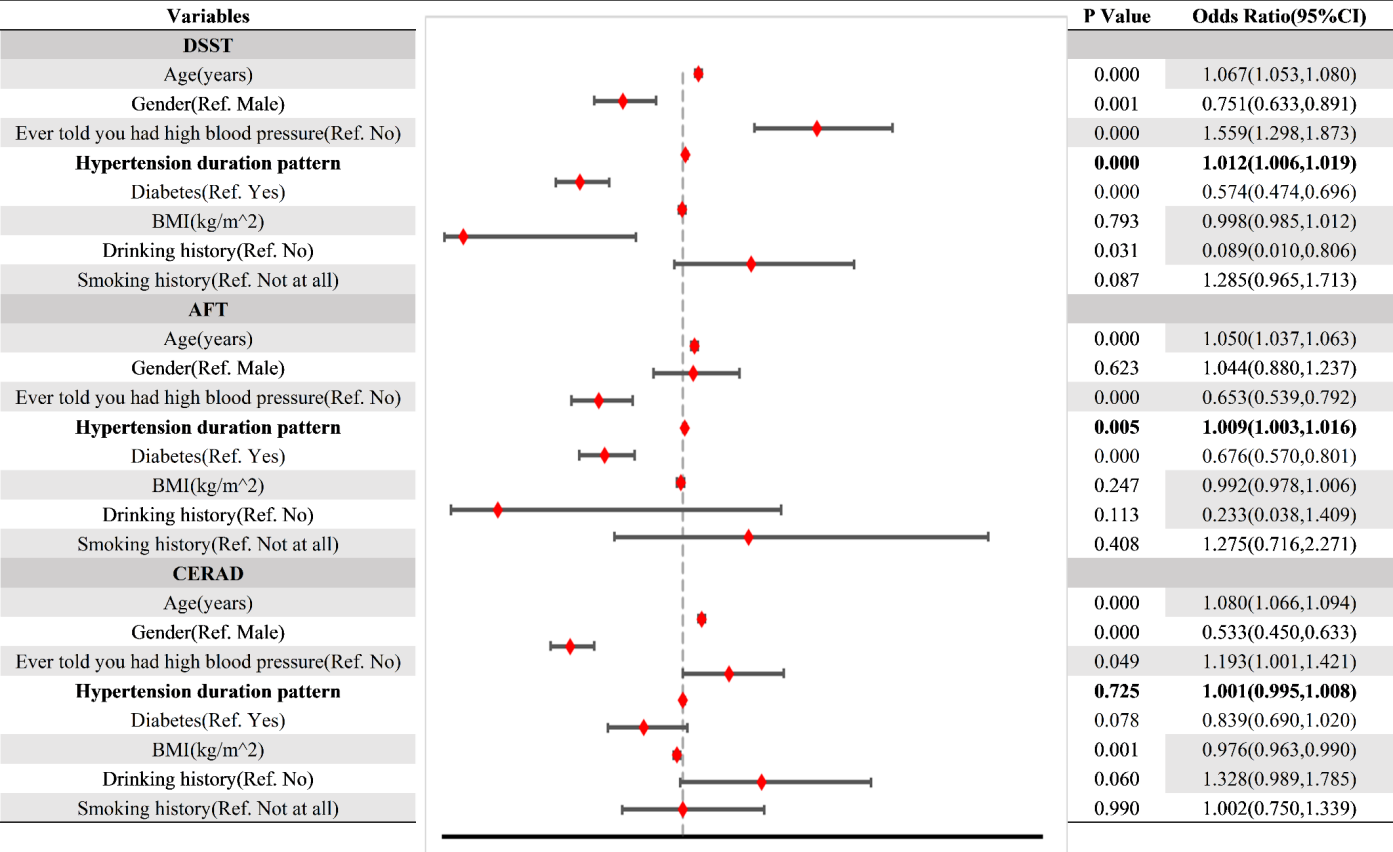
Identification of the risk factors between hypertension duration and cognition impairment. (DSST<32 scores; AFT<12 scores; CERAD test ≤ 20 scores)

Logistic regression models were used to assess the relationships between risky factors and cognition performance across all participants in Fig. 2. For the DSST, the analysis revealed that the older individuals exhibited the higher odds ratios for cognitive impairment, with an odds ratio (OR) of 1.067 (95% confidence interval: 1.053–1.080). A similar pattern was observed in the AFT, where the OR was 1.050 (95% CI: 1.037–1.063). However, no association was observed between the duration of exposure to hypertension and cognition performance in CERAD (*p* = 0.725). And for the other variables, non-diabetic individuals can significantly decrease the risk of DSST (OR 0.574 [95%CI 0.474–0.696]) and AFT (OR 0.676 [95%CI 0.570–0.801]) in the logistic regression model. Besides, females showed the lower OR for cognitive impairment, which was 0.751 (95% CI: 0.633–0.891) for DSST and 0.533 (95% CI: 0.450–0.633) for CERAD compared to the male populations.

### The duration-response analysis of hypertension with cognition function

**Fig. 3:**
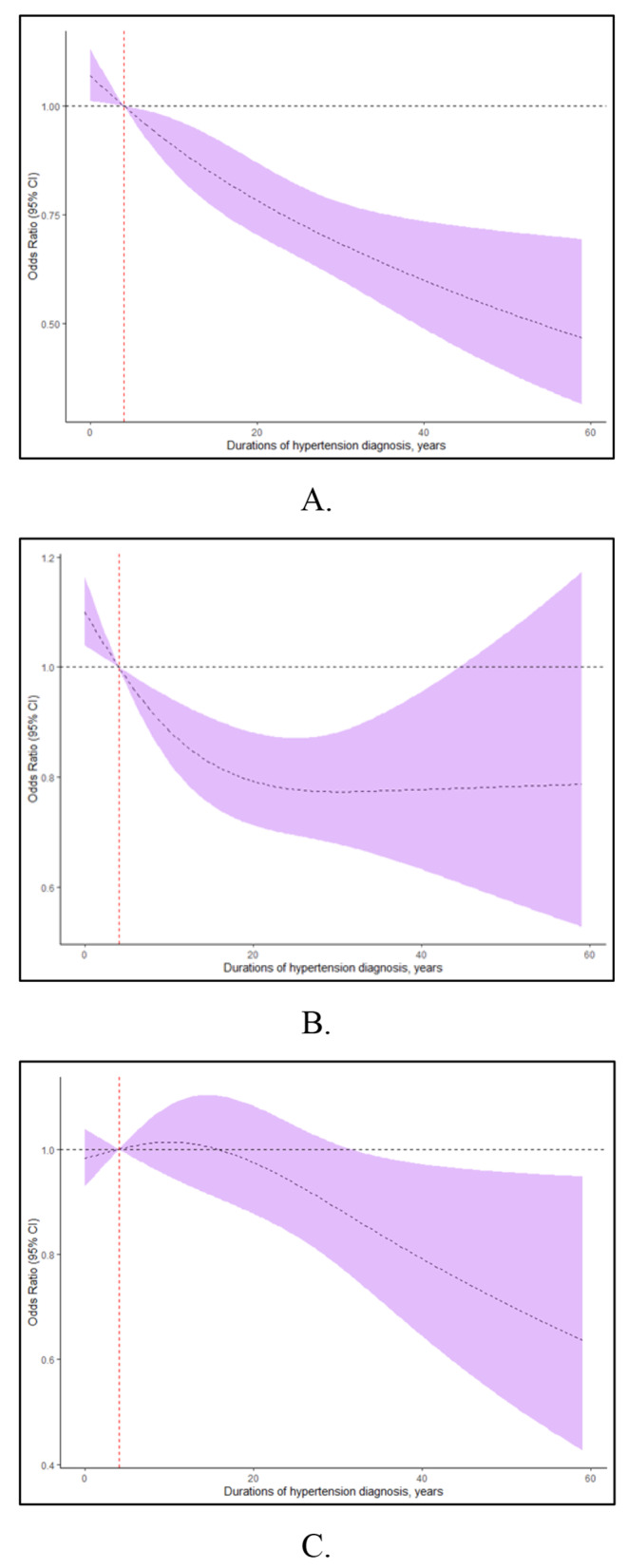
Relationship between the duration of hypertension diagnosis and risk of cognitive impairment. The model was based on restricted cubic spline (RCS) regression models (**A** for DSST; **B** for AFT scores and **C** for CERAD test). The results represent the estimated ORs and their 95% confidence intervals (OR, odds ratio)

Subsequently, the RCS analyses were applied to explore the duration-response relationship between hypertension and cognition function, and the results were presented in Fig. 3. The duration-response relationships between hypertension and cognition function (DSST and AFT) were aligned with the logistic model, showing significant results (*p*<0.050). Furthermore, the RCS analyses revealed a nonlinear association between the hypertension duration and cognitive function (AFT), with *P* value for nonlinear of 0.036. (Fig. 3) However, the RCS model demonstrated that longer hypertension duration was related to a decreased DSST cognitive score, with *p*-values for non-linearity of 0.758, respectively. (Fig. 3)

### Subgroup analyses of the association between hypertension duration and cognition function by RCS regression

Upper results revealed that middle-aged and older adult hypertension predicted different effects on the cognition performance. And incident hypertension suggests faster cognitive decline in middle-aged individuals. Thus, we further to detect the association between the duration of hypertension and cognition function among the middle-aged and older adult hypertension. An RCS model was used to evaluate the associations between the duration of hypertension and cognitive function. And we found that hypertension duration was reversely linear associated with CERAD scores (nonlinear *p* = 0.849) among middle-aged hypertension adults. (Fig. 4A) But the trend of CERAD scores was nonlinear for the older hypertension adults (nonlinear *p* = 0.013). (Fig. 4B) Likewise, RCS analysis exhibited a reverse linear association between the hypertension duration and DSST scores (nonlinear *p* = 0.391) for midlife hypertension participants. (Fig. 4C) And Fig. 4D revealed the non-linear relationship in DSST scores between the hypertension duration and cognition function (nonlinear *p* = 0.013) for the older hypertension. In Fig. 4E, RCS characterized no significance association between the hypertension duration and AFT scores (*p* = 0.539) for the midlife hypertension individuals. However, a significant nonlinear relationship was identified between the duration of hypertension and AFT scores in older groups (p for nonlinear = 0.014). (Fig. 4F)

**Figure:**
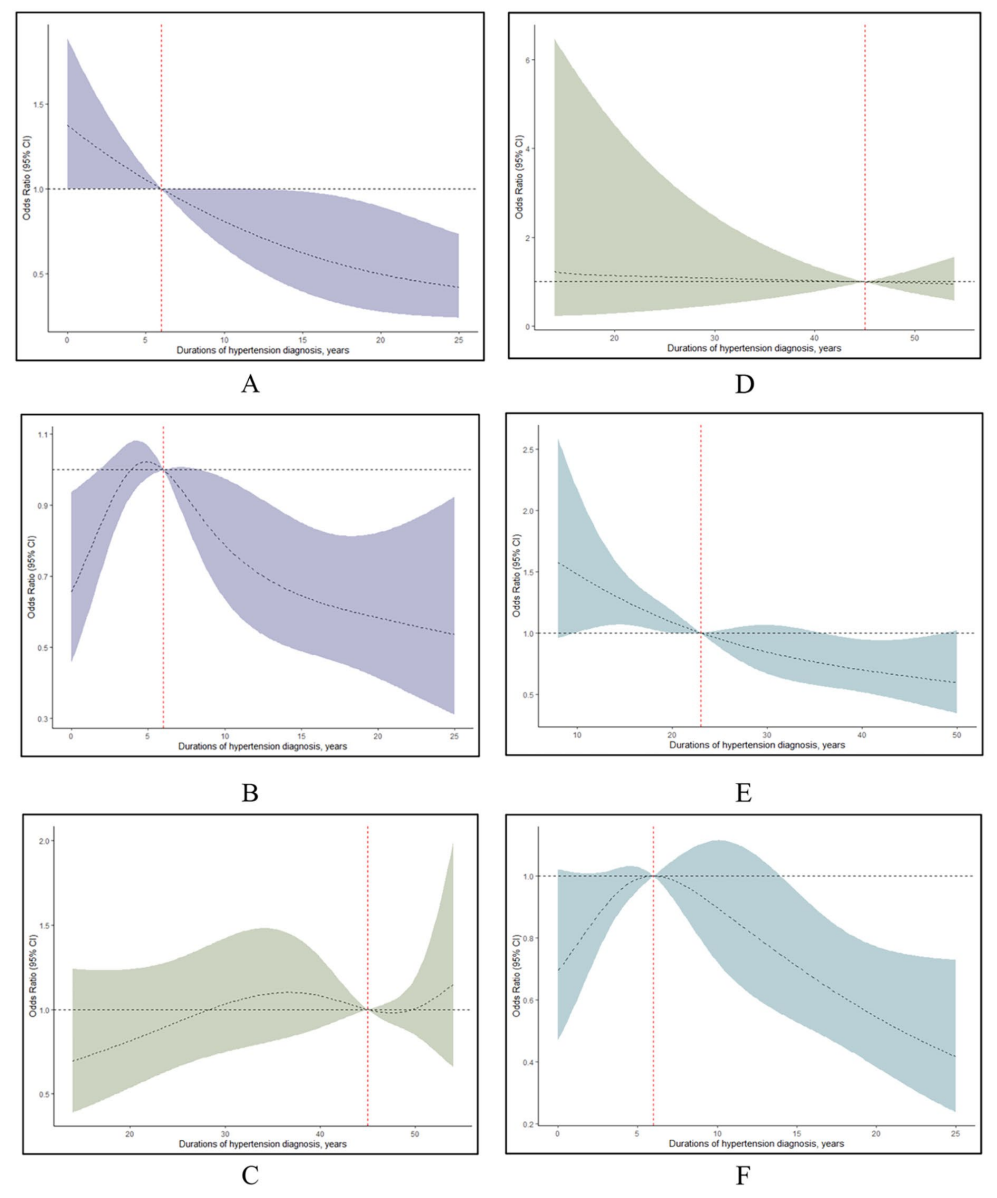
Subgroup analysis of the hypertension duration-response associations with cognitive impairment. The midlife (**A**, **C** and **E** < 55 years) and late (**B**, **D** and **F** ≥ 55 years) hypertension (**A** and **B** for DSST; **C** and **D** for AFT scores; **E** and **F** for CERAD test)

## Discussion

The duration of hypertension represents a significant indicator of cognitive performance [20, 22, 23]. Previous studies have demonstrated the relationship between hypertension and cognitive performance [13, 20, 21]. However, the conclusion from some cohort study of the relationship between duration of hypertension and cognitive performance was inconsistent. To the best of our knowledge, this cross-sectional study represents the reconfirmed exploration of the association between hypertension duration pattern and cognitive performance. And we discovered longer duration of hypertension was associated with the impairment of cognition, especially for middle-aged individuals.

Our findings revealed that the hypertension duration pattern was associated with the cognitive performance. Accordingly, the duration of hypertension is a critical factor in cognitive decline [20, 21]. Studies have shown that the longer an individual has hypertension, the greater their risk of experiencing cognitive decline. The correlations may be attributed to the complicated damage to the vascular system and the subsequent impairment of brain function [24, 25]. However, the age of incident hypertension onset does not seem to have directly effects on cognitive function. Our results are consistent with several previous studies of the relationship between hypertension duration and cognitive decline. A prospective cohort study found that increasing duration since hypertension initiation predicted lower mean cognitive z-score irrespective to the age of hypertension onset [20]. Besides, the evidences from visits 1 (2008–2010) and 2 (2012–2014) of ELSA-Brasil study indicated that hypertension were associated with faster cognitive decline, but the duration of hypertension diagnosis was not related to the cognitive impairment [21]. Those inconsistent studies have adopted various tools and methods to assess cognitive function, which may have effects on the comparable results. Besides, for the elderly population, survival bias and attrition may explain some inconsistencies. That is, individuals with poorer cognitive function may die earlier, thus influencing the study results.

In line with other studies, our results demonstrated the duration of hypertension diagnoses predicted linear cognitive decline among the midlife hypertension populations. Results from the Maastricht Aging Study (MAAS) showed that incident hypertension implied cognitive decline in middle-aged individuals [26]. Another research from the prospective Framingham Offspring Cohort Study Hypertension in midlife also concluded that midlife hypertension was associated with accelerated white matter hyperintensity volume (WMHV) progression (*p*<0.001) and worsening executive function (TrB-A score; *p*<0.012) [27]. Hypertension in middle age predicted significantly declines in cognitive functions which may be due to the more vulnerable to vascular damage during middle age [28–30]. Besides, middle-aged hypertension usually alongside with other cardiovascular risk factors, such as high cholesterol, diabetes, and smoking, and the cumulative effect of these factors can lead to cognitive decline state [17, 31, 32]. 

Respectively, our research findings revealed a positive relationship between the duration of hypertension and cognitive impairment. Importantly, optimal and effective interventions for hypertension can significantly reduce the risk of target organ damage [1]. In clinical practice, the focus of hypertension-mediated organ damage (HMOD) has been mainly on kidney damage, myocardial infarction, and other prevalent complications [1, 2]. Regarding vascular cognitive impairment, increasing evidences suggest that hypertension usually acts a pivotal deleterious factor in the development of cognitive impairment, which is often co-morbidities with neurodegenerative processes [33, 34]. Therefore, hypertension related damage of cognitive function may be recognized as a HMOD. Although, our study solely focused on the duration of hypertension as a cognitive impairment risk factor. However, the prolonged duration of hypertensive condition, along with the use of anti-hypertension medications and other treatment modalities may impose psychological burdens that could further impair cognitive function. Thereby, in clinical practices, it is recommended to perform vascular cognitive impairment neuroimaging with MRI to screen for hypertension-mediated cerebrovascular injury and to assess cognitive impairment. Subsequently, besides the well-established treatment algorithm for hypertension, the drug treatment strategy for patients with longstanding hypertension may require more specific modifications, for example the optimal combinations of cognitive impairment treatments. It means that health professionals also have responsibilities to verify the rules and regulations applicable to antihypertension drugs by the concomitant prescription that enhance cognitive function, including cholinesterase inhibitors, glutamate receptor antagonists, and neuroprotective compounds, preferably administered as early as possible.

Our study has some limitations that should be acknowledged. Firstly, the study was a cross-sectional design, which may lead to the cognitive assessment bias. And the enrolled populations were relatively old, which we cannot conduct a comprehensive study based on any age spectrum. Secondly, the diagnosis of cognitive impairment using the scores assessment may be less of systematic, and more solid methods need to be exploitative. Furthermore, the analysis did not consider the use of cognitive prescription due to the limited data, which is a potential confounding factor to influence the cognitive performance. Therefore, the results of this study need to be further investigated to validate these conclusions.

## Conclusion

In summary, we concluded that the increased hypertension duration patterns are associated to a greater cognitive impairment, particularly in middle-aged hypertension. Therefore, it is necessary for the earlier age of hypertension onset individuals to identify the causes of hypertension especially for secondary hypertension, and cure hypertension, in order to guard against cognitive impairment.

## Data Availability

No datasets were generated or analysed during the current study.
